# When the microbiome meets One Health principle: Leading to the Holy Grail of biology and contributing to overall well‐being and social sustainability

**DOI:** 10.1002/imo2.30

**Published:** 2024-09-10

**Authors:** Yazhou Zhou, Ziying Xu, Huan Zhang, Teng Liu, Jin Zhou, Yujing Bi, Yanping Han, Yafang Tan, Jing Yuan, Ruifu Yang

**Affiliations:** ^1^ State Key Laboratory of Pathogen and Biosecurity Academy of Military Medical Sciences Beijing China; ^2^ Department of Bacteriology Capital Institute of Pediatrics Beijing China; ^3^ School of Public Health Hebei Medical University Shijiazhuang China

**Keywords:** balance of dynamic factors, eco‐health, microbiome, One Health, precision medicine, sustainability

## Abstract

The convergence of microbiome science with the One Health principle heralds a transformative era in biology, prioritizing the collective well‐being of humans, animals, and the environment. This review delves into the intricate dance between microbiomes and their hosts, revealing their profound impact on health, nutrient cycles, and climate change. Championing a unified approach to health issues across diverse kingdoms of life, One Health emerges as a holistic strategy, underscored by a proposed universal balance theory, “Balance of Dynamic Factors.” This theory spotlights the equilibrium within microbial and human‐animal‐environment interactions, offering a revolutionary pathway to global health and social well‐being. It paves the way for disease prevention, health equity, and sustainability, all of which are purviews of a balanced ecological system. We navigate the challenges and opportunities of this integrative approach, culminating in a call for action for the incorporation of microbiome science into health policies, precision medicine, legislation, eco‐health projects, and education, thereby setting the stage for harmonious coexistence with our planet.

## Highlights

- The microbiome is key for interdisciplinary solutions to global health issues.
- A systems‐thinking approach to disease surveillance and environmental care should be adopted.
- The microbiome and One Health should be integrated to understand life complexity and shape health policies.

## Graphical abstract

Unveiling the microbiome as a cornerstone for interdisciplinary solutions to global health challenges, this theory integrates microbiome research with the One Health approach to develop innovative strategies for disease surveillance, environmental stewardship, and public health interventions. By embracing a systems‐thinking perspective, it navigates the complexities and opportunities presented by a universal balance theory, advocating for the integration of microbiome insights into global health policies. This holistic approach seeks to ignite a paradigm shift, fostering a comprehensive and unified strategy that not only enhances global well‐being but also promotes long‐term sustainability and resilience against emerging health threats.

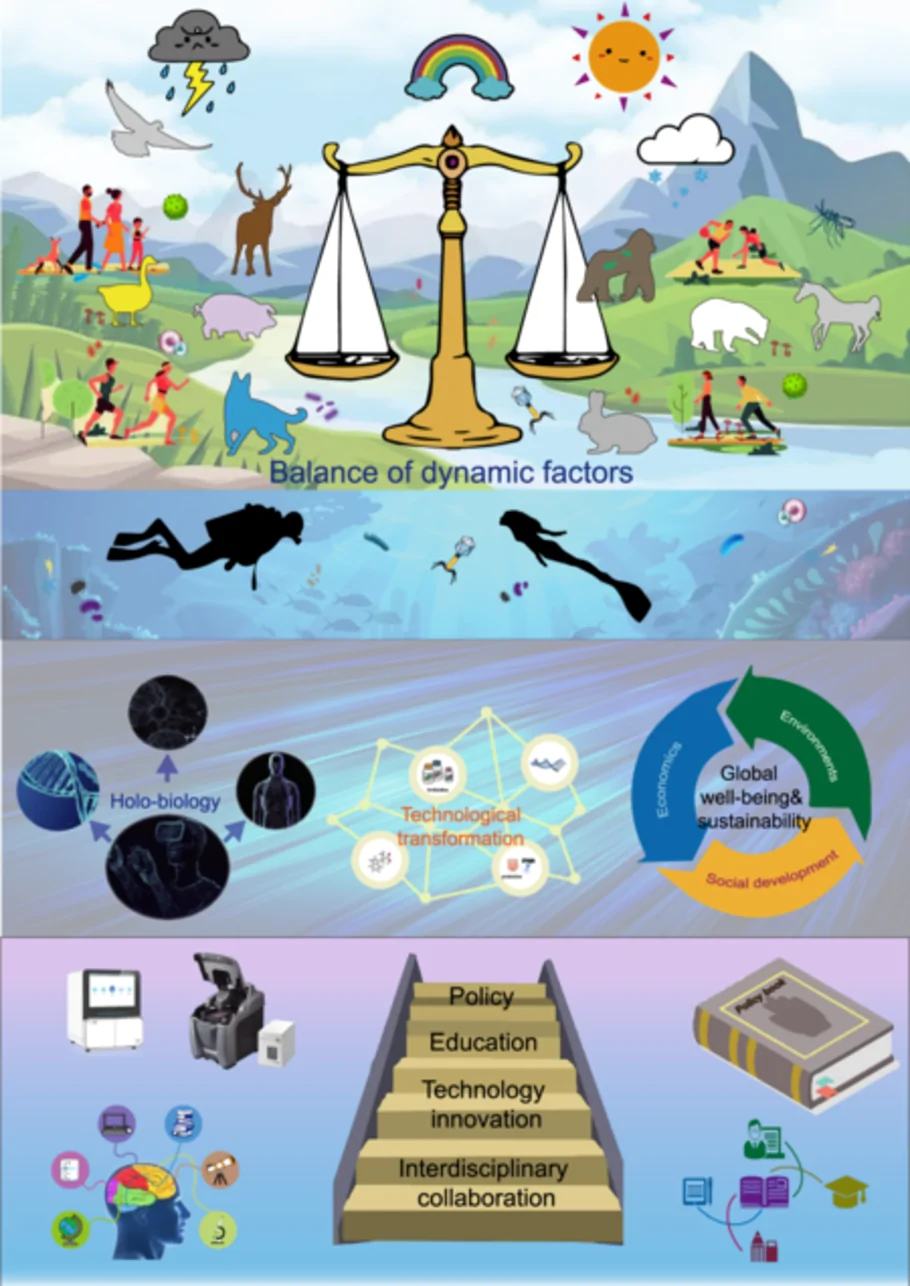

## 1 INTRODUCTION

Deeply ingrained in our minds is the profound question [1]: what does biology truly encompass? The past century has witnessed a transformative journey in biological understanding, progressing from the study of individuals to populations [2], from phenotypes to genotypes, and from single pathways to complex systems [3]. Despite the emergence of diverse biological disciplines, each seeking to deepen our comprehension of the natural world, there remains a fundamental challenge: how can we universally and systematically unveil the intricate biology woven within all life forms on our diminutive planet?

**Figure 1:**
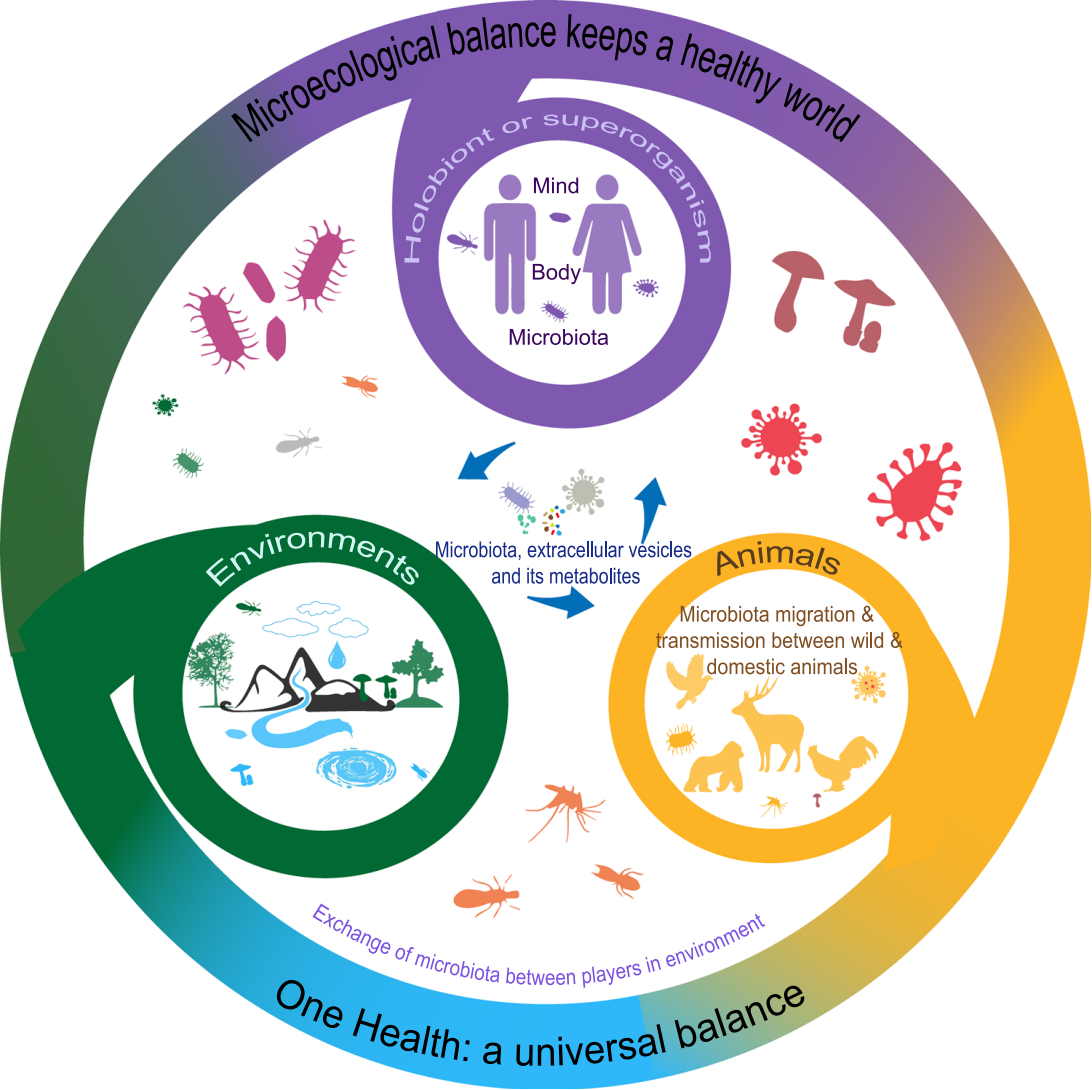
The microbiome plays a crucial role as a bridge in the interactions and interdependencies among humans, animals, and the environment. As superorganisms or holobionts, the interplay and balance between the body inherited from parents, the mind, and the microbiome determine the health and occurrence and development of diseases in humans. Animals and the environment form a balanced system with their microbiome. These “individual” microecological balance systems influence each other, with the microbiome acting as a bridge, facilitating an inseparable close interaction from the communication of the microbes themselves to the interregulation at the molecular level. This constitutes a balanced system of humans, animals, the environment, and the microbiome. The maintenance of this system's balance is the goal pursued by One Health, which aims to benefit all things and maintain a healthy globe.

The swift progression of DNA sequencing technologies [4] has been a catalyst in overcoming this challenge, shedding light on the once‐overlooked realm of microbes and elucidating their indispensable roles in sustaining the well‐being of humans, animals, plants, and our planet as a whole—extending even to the vast cosmos [5]. This enormous empire of microscopic entities, collectively known as the microbiome or microbiota, comprises bacteria, viruses, fungi, protists [6], archaea, and more [7]. Intimately intertwined with their hosts and environments, these entities intricately determine the vitality of their respective niches (Figure 1). Most research has focused on bacteria and their functions in the human microbiota. A recent study revealed that the distribution of the gut eukaryotic genus *Blastocystis* is associated with dietary habits and contributes to cardiovascular metabolic health [6]. Taking the example of the human gut [8], we uncover a microcosm that harbors millions of these minuscule beings, forming a complex ecosystem through intricate interactions among themselves and with their human hosts. These findings reveal their critical role in maintaining human health and their close association with the development of diseases [9, 10, 11].

Moreover, diverse environments, such as the ocean, make paramount contributions by microorganisms to essential carbon, nitrogen, and sulfur metabolic processes [12]. Soil, which functions as a vital living ecosystem with a high microbiome, sustains plants, animals, and humans and plays a key role in water quality, climate change, and human health [13]. Climate change, deforestation, flooding, or drought will result in outbreaks of vector‐borne diseases, such as malaria, Zika, dengue, and Lyme disease [14], the spread of highly pathogenic avian influenza by altering bird habitat ranges and migration patterns [15], the emergence of novel pathogenic pathogens [16], and the destabilization of fungal‐bacterial interactions that impact biodiversity [17]. The microbiome could play a critical role in addressing the issues of global change and sustainability [18]. Climate‐driven microbiome dynamics can be used for assessing an ecosystem's vulnerability to environmental perturbations, and the microbiome can generate metabolic help to handle environmental pollutants, such as the breakdown of plastics [19]. The dynamic ecosystem of the gut microbiota also plays a vital role in animal health. Maintaining this balance is essential for preventing disease and reducing the reliance on antibiotics, which is crucial in the global effort to combat antibiotic resistance. The rapidly evolving field of microbial ecology offers promising alternatives for sustainable animal husbandry practices [20].

## 2 MICROBIOME: THE INDISPENSABLE GENOME FOR CREATURES AND A KEY PLAYER IN THE ENVIRONMENT AND ECOSYSTEMS

### The omnipresent microbiome

The microbiome refers to the diverse community of microorganisms that inhabit various environments, such as the human body, animals, soil, water, and air. These microorganisms, which constitute a dynamic and interactive community, play crucial roles in maintaining the health and functioning of their respective ecosystems, often regarded as the second genome for creatures [21]. Therefore, the microbiota plays a pivotal role in shaping the landscape of biology (Figure 1). The multifaceted nature of the microbiome represents its importance in the intricate dance of life and the maintenance of health across various life forms. Each component contributes uniquely to the overall functionality of this second but indispensable genome, and each component also has a complex interaction with the host. It acts as a ‘silent’ orchestrator, influencing physiological processes and contributing to the overall well‐being of hosts. From the human gut microbiota, which influences nutrient absorption and immune system development, to the soil microbiota, which supports plant growth and nutrient cycling, the microbiome is an indispensable player in the symphony of life. The understanding of microecological complexity has allowed scientists to explore the intricate world of microorganisms with unprecedented precision [22].

### The omnipotent microbiome

The awareness of the importance of the microbiome in human health has shaped our concept of humans in traditional medicine, that is, the human is a superorganism or holobiont that is composed of the traditional medical view of the human body, the mind, and the microbiome [23]. The microbiome exists almost everywhere in the human body [9]. The largest body of the microbiome inhabits the gut, which consists of trillions of microorganisms in the digestive tract and influences digestion, nutrient absorption, metabolism [24], and immune function [25]. *Oscillibacter* spps. encode some conserved cholesterol‐metabolizing enzymes, regulating lipid homeostasis and benefiting cardiovascular health [26]. Imbalances in the gut microbiota have been linked to various health conditions, including obesity, inflammatory bowel disease (IBD), and metabolic disorders [27]. The oral and skin microbiomes are key to protecting against pathogens, maintaining barrier function, and regulating immune responses [28, 29]. Their dysbiosis can contribute to different diseases, including acne, eczema, dermatitis, dental caries, periodontal diseases, and even cardiovascular diseases and diabetes [28, 29]. A recent report revealed the composition of strains of *Cutibacterium acnes* in different skin ecological niches and delineated the functional characteristics of gene expression and metabolites of this bacterium in different diseases and skin sites, revealing the combined effects of genetic factors and the microenvironment on its function [30].

The gut microbiome of livestock influences digestion, nutrient utilization, and overall health [31, 32, 33]. Modulating the gut microbiota can improve feed efficiency, reduce disease susceptibility, and increase animal welfare in agriculture. Research on the microbiome of wildlife species has revealed insights into their adaptation to diverse habitats, immune responses, and conservation strategies [31, 33, 34, 35]. Understanding the role of the microbiome in wildlife health is crucial for biodiversity conservation efforts.

In addition, microbiomes play crucial roles in the health and well‐being of plants, contributing to their physiological metabolism and defense against pathogens through the production of antimicrobial compounds or by priming the plant's immune system. Microbiomes can increase the availability of nutrients for plants. Some members of the plant microbiome produce hormones that stimulate plant growth [36], such as auxins, cytokinins, and gibberellins. Members of the soil microbiome contribute to soil structure and fertility, which in turn benefits plant growth [37, 38, 39]. They decompose organic matter and recycle nutrients, making them available for plants. Understanding and harnessing the power of plant and soil microbiomes can lead to more sustainable agricultural practices, reducing the reliance on chemical fertilizers and pesticides [39]. The aquatic microbiomes in both freshwater and marine environments play roles in water quality, nutrient cycling, and microbial community dynamics [40]. Monitoring and managing water microbiota are essential for safeguarding aquatic ecosystems and human health. Sustainable agricultural practices leverage soil and water microbiota for improved crop yields, soil health, environmental sustainability, and ecosystem resilience. The air microbiome significantly impacts the gut, skin, and respiratory microbiota of humans and animals [41, 42]. Additionally, the unique and complex microbial communities found in the troposphere are influenced by multiple environmental factors and may have potential implications for human and animal health [43].

## 3 ONE HEALTH: A CONCEPT TO UNITE ALL IN OUR GLOBE

### Multidisciplinary One Health

One Health approach is an interdisciplinary approach that recognizes the close interconnectedness of the health of people, animals, plants, and their shared environment [44]. It is based on the understanding that the well‐being of all these components is closely interconnected and interactive and that changes in one component can have ripple effects on the others, advocating for integrated efforts to address health challenges and promote overall well‐being.

Human health is intimately linked to the health of animals and the broader ecosystem. Zoonotic diseases, those transmitted between animals and humans, exemplify the need for a unified strategy [45]. The emergence of diseases, such as coronavirus disease 19 (COVID‐19), underscores how disruptions in natural balance can have far‐reaching consequences [46]. This also underscores the urgent need for One Health to break down traditional silos and foster collaboration among diverse disciplines to address health challenges at the intersection of species and ecosystems [47]. In addition to emerging infectious diseases, antimicrobial resistance, environmental pollution, and ecological degradation also require a collective response that considers the intricate web of interactions within ecosystems [48, 49]. A systems‐thinking approach positions One Health itself as a crucial framework for mitigating the impacts of global health threats [50].

### Cross‐sector integrated One Health

The core principles of the One Health approach include interdisciplinary collaboration, systems thinking, preventive strategies, and global collaboration [51]. Collaboration among various disciplines, such as medicine, veterinary science, environmental science, public health, and social sciences, will foster a comprehensive understanding of health issues and facilitate the development of holistic solutions [52]. A systems‐thinking perspective recognizes the complex interactions and feedback loops within ecosystems. It considers the social, economic, and environmental determinants of health, addressing underlying factors that influence health outcomes [53]. Preventive measures for mitigating health risks and preventing the emergence and spread of diseases include surveillance, early detection, vaccination programs, antimicrobial stewardship, and environmental conservation efforts. Global cooperation and partnerships for tackling global health challenges, infectious disease outbreaks, antimicrobial resistance, zoonotic diseases, and environmental degradation stress the importance of shared responsibility and coordinated action at the local, national, and international levels (Figure 1). The implementation of One Health approaches helps us in disease control and prevention, environmental conservation, food security and safety, and social equity and resilience, promoting human well‐being and social sustainability [54].

### Examples calling for one health

**Figure 2:**
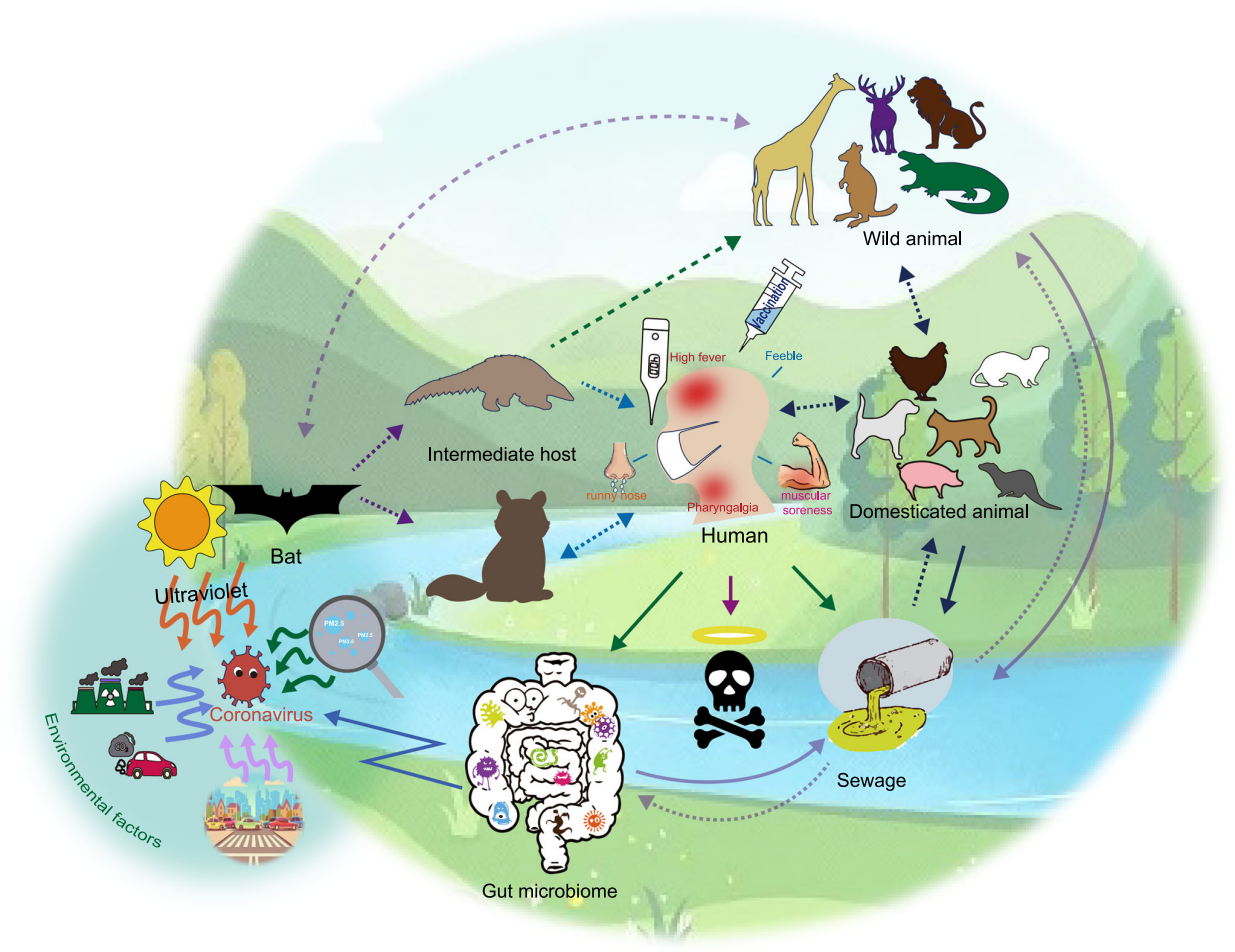
The global pandemic of coronavirus disease 2019 (COVID‐19) has reminded us of the significant value of the One Health strategy when dealing with infectious diseases and other global crises. The possible hosts considered for this novel coronavirus are bats. The virus spreads to humans through probable intermediate hosts (such as pangolins or other animals), and it may also spread to other wild animals. Infected humans may also transmit the virus to domestic animals or wild animals. The virus excreted from the intestines of humans and animals can cause new transmission through contaminated water sources or sewage. The occurrence and spread of this novel coronavirus can also be influenced by natural environmental factors, such as temperature, climate, haze, and PM2.5 particulates, which can affect the spread efficacy of the virus. The prevention and control measures taken by humans, such as the closure of transportation, the interruption of personnel exchanges, and the immunization of vaccines, can also affect the spread and variation of the virus. The emergence, spread, prevention, and control of the novel coronavirus require the cooperation of multiple disciplines, including microbiology, epidemiology, zoology, immunology, ecology, genomics, evolutionary biology, bioinformatics, and so forth. Policy formulation and implementation by decision‐makers and, more importantly, a united response from the international community is also needed.

The COVID‐19 pandemic has underscored the need for a holistic and cooperative plan, implementing the One Health approach, to effectively address infectious diseases and other worldwide issues [55] (Figure 2). A major lesson for policymakers is that more knowledgeable decisions should be made [56], which not only safeguard human health but also consider the health and welfare of all other living beings on our interconnected planet, Earth. Environmental contamination from medical and animal waste fuels soil‐based antibiotic resistance, risking human health through microbiome alteration and resistant bacteria growth. Antibiotic use and resistance spanning the human, animal, and environmental sectors urgently require a unified One Health strategy with robust surveillance and action plans that are vital for preventing antibiotic resistance and promoting global health harmony [57].

The use of antibiotics and their impacts on environmental, human, and animal health are typical examples of the urgent need for a combination of OneHealth strategies and the microbiome [58]. Anthropogenic antibiotics are increasingly released into soil and water environments, potentially altering microbial communities and leading to the selection of antibiotic‐resistant microorganisms, including animal and human pathogens. Although planktonic microalgae, fungi, or plants in the environment have the capacity to biosorb, photodegrade, or detoxify antibiotics, a multidisciplinary effort should be strengthened to develop the molecular ecology of antibiotics and to understand the reactive transport of antibiotic molecules [58], promoting appropriate environmental interventions to reduce microbial resistance to antibiotics.

Fungi that are harmful can lead to illnesses in both the animal kingdom and human society, as well as in the plant world. They have the ability to endure for extended periods and propagate through the release of spores. The spotlight in this instance is on the organism *Coccidioides immitis*, which is a disease‐causing agent for both humans and animals and can thrive in soil [59]. This serves as a prime example of the necessity for an integrated One Health strategy for developing preventative measures.

The examples above indicate that One Health is crucial for addressing cross‐sector health threats, fostering global cooperation, and ensuring the well‐being of humans, animals, and the environment [56].

## 4 MICROBIOME MEETS ONE‐HEALTH PRINCIPLES: A UNIVERSAL BALANCE THEORY FOR OVERALL HEALTH AND SOCIAL SUSTAINABILITY

### Science behind One Health

The convergence of the microbiome and the One Health principles ushers in a paradigm shift that redefines our understanding of life and environments. We need to rethink the science behind One Health [52], which is multifaceted and involves multiple disciplines, including veterinary medicine, human medicine, environmental science, epidemiology, and ecology. At its core, One Health science aims to understand the complex interactions between different biological systems and how they influence each other through shared environmental factors. Considering the numerous interacting factors that influence the overall health of a system, including biological, environmental, social, economic, and political determinants [25, 60], a universal theory, the balance of dynamic factors, is proposed for interdisciplinary One Health (Table 1). This theory seeks equilibrium and resilience in microbial ecosystems and human–environment interactions [53, 61, 62]. A balance needs to be achieved among the above factors to maintain the well‐being of the system. In essence, this interactive balance acknowledges the symbiotic relationship between self‐genes and microbiome genes, paving the way for a more holistic understanding of living organisms and ecosystems. For example, in the context of zoonotic diseases [49, 63, 64], the balance of factors, such as animal health, human health, environmental conditions, and social and economic factors all play a role in preventing the emergence and transmission of these diseases [65].

**Table 1:** Foundation and principles for the universal balance theory “balance of dynamic factors”.

Foundation	Principles	Key components
Interconnectedness: complex microbial ecosystems	The interconnected microbial ecosystems across humans, animals, and the environment shape health outcomes and ecosystem dynamics.	Human, animal, and environmental microbiomes: influencing health, immunity, and functions in these components.
Balance and Resilience: homeostasis across systems	Maintaining a microbiome equilibrium is essential for health resilience, disease prevention, and ecosystem sustainability.	Microbial diversity, host‐microbiome interactions, and ecological balance: enhancing ecosystem resilience and sustainability.
Harmony in health: microbiome‐host coevolution	Coevolutionary relationships between hosts and microbes shape health adaptations, immune system development, and disease tolerance.	Microbial symbiosis and adaptations as well as host‐microbiome crosstalk: promoting health outcomes and maintaining symbiotic balance.
Adaptive responses: microbiome plasticity and flexibility	Microbiomes exhibit plasticity and adaptability to environmental changes, host factors, and microbial community shifts, influencing health outcomes.	Microbiome dynamics, resilience and restoration: ecosystem adaptability and health maintenance, microbial‐based therapeutics.
Sustainable practices: promoting health and ecosystem integrity	Sustainable practices, informed by One Health principles and microbiome science, support health equity, environmental conservation, and social well‐being.	One Health strategies, health equity, and environmental stewardship: promoting holistic solutions to global challenges, enhancing health equity and community resilience, and supporting ecosystem integrity and health sustainability.
Knowledge integration: translating research into action	Knowledge integration, evidence‐based practices, and stakeholder engagement facilitate the translation of microbiome research into actionable interventions and policy recommendations.	Research collaboration, policy alignment, and public engagement: enhancing innovation and best practices, integrating microbiome considerations into different sectors; promoting sustainable practices for health and sustainability.

### Rethinking the life pattern

When the holistic approach of One Health is coupled with the microbiome perspective, it forms a synergistic framework that considers the microbial dimension in the intricate web of life [66, 67]. This integration expands the scope of One Health, rendering the microbial community key and interwoven players in understanding living organisms and ecosystems [68]. Traditional biology often separates the study of individual organisms from their environments. However, the integrated approach catalyzed by the microbiome and One Health calls for a shift toward a more holistic understanding. Organisms, including humans, are not isolated entities but integral components of complex ecosystems shaped by intricate interactions with their microbiomes [49, 52, 69]. In terms of genes, the traditional focus on the host organism's genetic material should be broadened to include the symbiotic genetic repertoire of the microbiome. This recognition reveals a dynamic interplay where host and microbial genes coalesce, influencing each other in ways that transcend conventional genetic paradigms [69, 70]. This symbiotic relationship redefines the boundaries of traditional biology, underscoring the need for an integrated approach that considers collective genetic contributions to health and well‐being [66].

**Figure 3:**
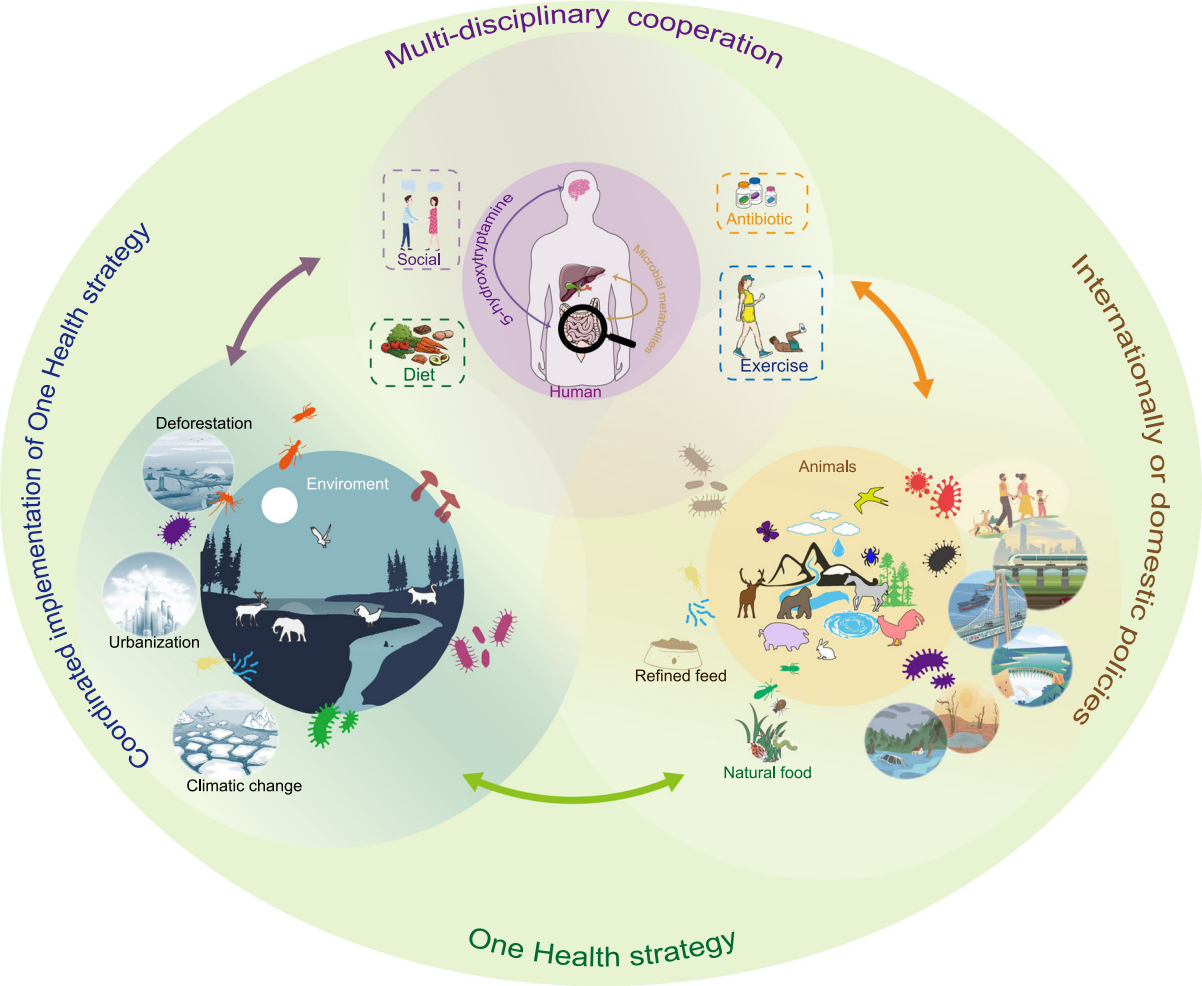
The health of humans, animals, and the environment are inextricably linked. The human microbiota is influenced by diet, drugs, social activity, and exercise. Human activities, including the construction of reservoir dams, the construction of railways and bridges, animal communities, natural foods, and refined feed, impact the microbiological balance of animals. Climate change, urbanization, and deforestation greatly influence the environmental equilibrium. All these mentioned or unmentioned factors interact closely with each other to maintain dynamic balance.

The synthesis of microbiome research and One Health principles is grounded in the recognition that the health of humans, animals, and the environment are inextricably linked (Figure 3). The amalgamation of microbiome research with One Health principles promises a future where health interventions are more holistic, surveillance systems are more proactive, and policies are better equipped to navigate the complex web of health, disease, and sustainability. The potential synergies between microbiome research and One Health principles include [53, 54, 68]: (1) promoting interdisciplinary collaboration for bridging gaps between disciplines, fostering knowledge exchange, and fostering innovative solutions that consider the interconnectedness of microbial ecosystems, host health, and environmental factors; (2) building eco‐health and ecosystem resilience by understanding microbial ecosystem services, ecological interactions, and ecosystem health indicators, informing conservation strategies, land management practices, and biodiversity conservation efforts; (3) developing disease surveillance and early warning systems for improving preparedness, response coordination, and public health interventions for zoonotic diseases, antimicrobial resistance, environmental hazards, and infectious disease outbreaks; and (4) fostering public health interventions and policy recommendations for supporting data‐driven decision‐making that considers health equity and environmental sustainability.

### Challenges and strategies

However, challenges and limitations exist in implementing the universal balance theory, including technological barriers, policy complexities, societal attitudes and behaviors, research and knowledge gaps, and resource constraints (Table 2). Collaborative efforts, innovative solutions, policy reforms, public engagement, and investment in research, technology, and education are required to address these challenges and limitations (Table 2).

**Table 2:** Challenges and limitations in implementing a universal balance theory and strategies for overcoming challenges and advancing research.

Challenges and limitations	Key factors	Strategies
Technological barriers	Data integration Computational complexity Technological accessibility	Technological advancements: Invest in bioinformatics Develop data standards Promote open science Harness AI and machine learning
Policy complexities	Regulatory frameworks Cross‐sectoral coordination Global governance	Policy and governance: Multisectoral coordination Regulatory harmonization Global health diplomacy
Societal attitudes and behaviors	Public awareness Cultural beliefs Behavioral change	Public engagement and education: Health literacy programs Behavioral interventions Citizen science
Research and knowledge gaps	Scientific complexity Data gaps Translational research	Research and innovation: Longitudinal studies Translational research Interdisciplinary collaboration
Resource constraints	Funding challenges Infrastructure needs Capacity building	Capacity building and investment: Training programs Investment in infrastructure Public‒private partnerships

For future integration of microbiome science and One Health principles into public health policies and practices, we need to implement the following actions: (1) Microbiome surveillance programs and One Health data platforms are needed to integrate all microbiome data, epidemiological information, environmental factors, and health outcomes to facilitate real‐time monitoring, early warning systems, and data‐driven decision‐making. For the integration of these two fields under one technical platform, we need to develop AI‐assisted bioinformatic techniques, including transomic data integration, analytical tools for different types of data, and AI‐assisted decision‐making systems. (2) Microbiome‐based diagnostics and microbial‐based therapeutics should be developed for precision medicine and personalized health. How to take full advantage of our current knowledge of microbiome dysbiosis and diseases to develop integrative diagnostic strategies for health evaluation and early disease diagnosis is a major challenge. (3) Health legislation and global health diplomacy should be advocated for promoting interdisciplinary collaboration and strengthening health capacity worldwide. This requires countries to negotiate international legislation or jointly abide by operational conventions under the framework of the World Health Organization (WHO) to promote international cooperation and joint actions under the integrated framework of One Health–Microbiome. (4) Eco‐health initiatives and One Health land use planning for integrating microbiome science and One Health principles to minimize environmental impacts, mitigate pollution, and preserve natural habitats. As mentioned above, only internationally coordinated actions can achieve sustainable development worldwide. (5) Public health education and awareness can be provided by conducting microbiome literacy programs and One Health advocacy for enhancing public understanding of the microbiome and promoting health equity, environmental stewardship, and social sustainability. Owing to the imbalance in world development, cultivating the literacy of people worldwide regarding the integration of the microbiome and One Health is an important task of public education. This is a great goal that can be achieved only under an internationally consistent framework. (6) Interdisciplinary research collaboration and the harness of emerging technologies for addressing complex health challenges through integrated approaches and bridging knowledge gaps in microbial ecology, host‒microbe interactions, and ecosystem health. From basic research to advanced practical applications, such as AI, bioinformatics technology, omics technology, and integration technology of information from different dimensions, these determine the goals and processes of interdisciplinary research and determine the possibility of sustainable development worldwide. (7) Training programs and cross‐sectoral collaboration for capacity building and workforce development. Capacity building is not an issue for a single country or region but rather a great project that requires the joint promotion of the entire world. How to propose a set of training plans that can be universally shared worldwide and promote close cooperation in different fields is an important task for us to advance the theory and practice of the balance of all things.

Regrettably, as of now, there is no case that integrates the disciplines of microbiome, environment, humans, and animals together to achieve the well‐being of humans, animals, and people. We hereby have no choice but to propose the above principle‐based suggestions and look forward to the implementation of these suggestions to achieve the true integration of the microbiome and One Health. However, many interdisciplinary studies have shown some achievements at these intersections. For example, a recent metagenomic sequencing analysis of groundwater from the Tibetan Plateau revealed the prevalence of antibiotic‐resistant pathogens (ARPs) and their bacterial resistome, implying different health risks [71]. A recent report from collaborations among biologists, ecologists, statisticians, computer scientists, and historians demonstrated no evidence for persistent natural plague reservoirs in either historical or contemporary Europe because of soil texture and biochemistry and the low diversity of rodents [72].

## 5 HOW MICROBIOME AND ONE HEALTH PRINCIPLES SHAPE BIOLOGY IN THE FUTURE

### Rational thinking of the first principles of biology

**Figure 4:**
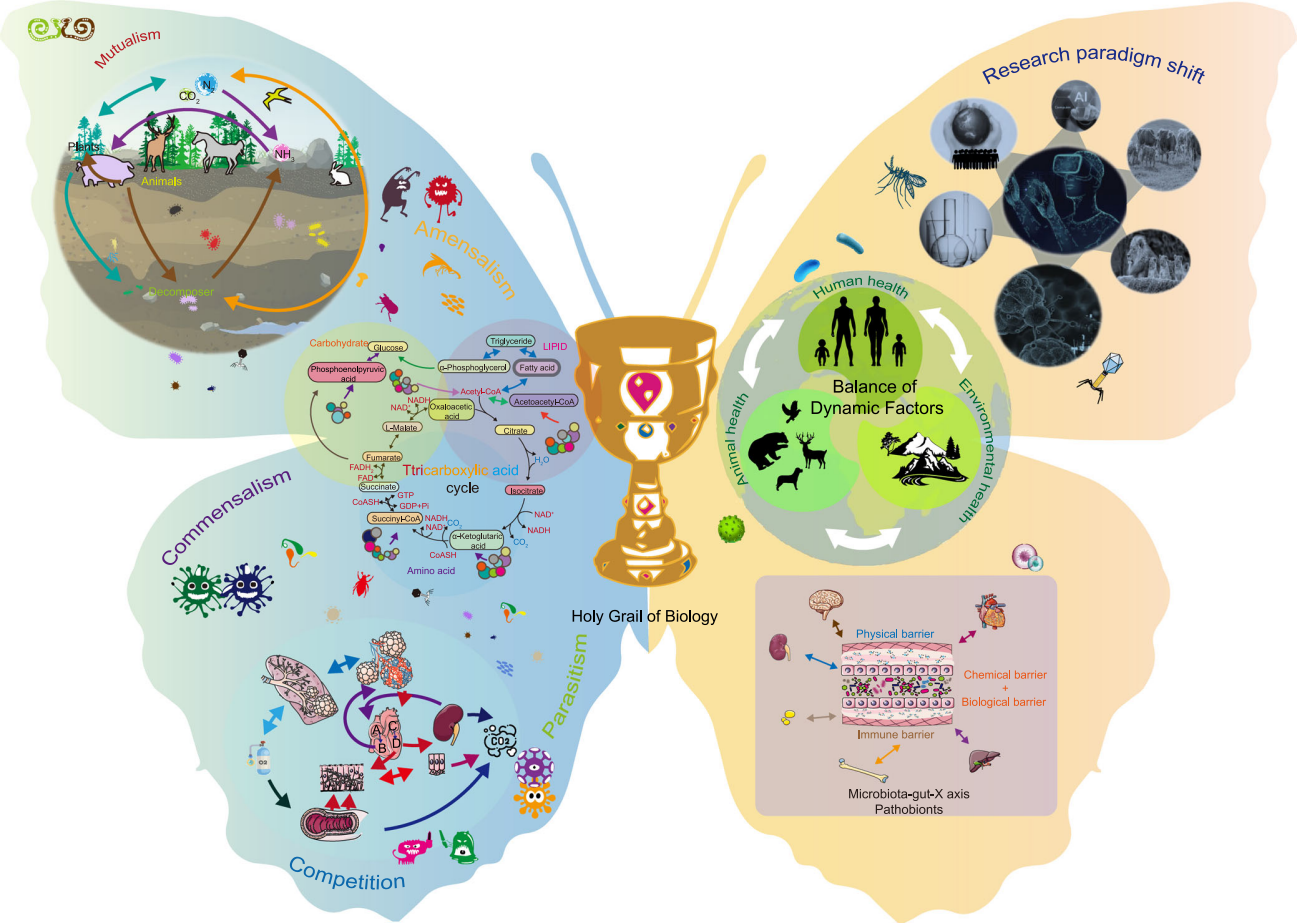
The integration of systems thinking of One Health with the microbiome laid the foundation for theoretical innovation in biology, ecology, medicine, and health, providing an opportunity for us to uncover the Holy Grail of Biology. Microecological exchanges between humans, animals, and environments through microbial players, their metabolites, and extracellular vehicles determine all well‐being on Earth (left wing). The integration of microbiota and the One Health arena will promote our research paradigm to shift from self‐focused research to a population‐ and ecology‐based framework (right wing). The “microbiota‐gut‐X axis” and “pathobiont” theories will impact the development of diagnostic and therapeutic technologies for diseases. The understanding of holobiont‐based evaluations for health and diseases should greatly impact health and disease management. The systemic way of thinking also profoundly impacts animal husbandry, wildlife, and ecology conservation. This has also prompted us to deeply consider the science behind One Health, the balance of dynamic factors or the balance of all things. One Health strategies call for coordinated international and domestic policies to uncover its science for developing technical strategies to balance the globe. Microbial players maintain dynamic equilibrium; the metabolic basis of this balance; and the core elements that determine the health of humans, animals, and the environment. This also helps us develop innovative techniques, policies, and international cooperation for implementing One Health strategies.

As mentioned in the introduction, the superorganism human is a symbiotic system with complex interactions between microbiota members and host organs that shape our health. The integration of systems thinking of One Health with microbiome research will inspire us to reconsider the principles of biology in the textbook (Figure 4). The first principles of biology can be understood as the fundamental and underlying concepts that form the basis of the study of life [73, 74]. These core first principles underpin the vast and complex field of biology. An organism is considered to be composed of one or more cells, and cells are the basic unit of life. For example, a single‐celled organism such as an ameba can carry out all the essential processes of life within one cell. Multicellular organisms, such as humans, are made up of countless specialized cells working together. Another important principle is the principle of evolution by natural selection. This explains how species change and adapt over time. An example is the evolution of the peppered moth. During the Industrial Revolution, darker‐colored moths were more likely to survive and reproduce, as they were better camouflaged against soot‐darkened trees, leading to a shift in the moth population. The principle of genetics is also fundamental. This process involves the inheritance and variation of traits through genes. We can observe this in how certain genetic disorders are passed on within families or how new traits arise through genetic mutations. The principle of homeostasis is also crucial. It refers to the ability of an organism to maintain a stable internal environment despite changes in the external environment. For example, our body regulates body temperature to stay within a narrow range regardless of the outside temperature. The primary principles of the aforementioned organisms are all considered from the perspective of the organisms themselves, whether at the cellular, genetic, evolutionary, or homeostatic level, and they all consider the composition and structure of the organisms themselves. Recognizing the microbiome as an essential component of living beings and environments [75], and based on the One Health principle [53], there is an inseparable and close connection between organisms, their microbiomes, and the environment. Therefore, when considering the first principles of biology, we must consider the close interactions among an organism's own genes, cells, metabolism, and microbiome. These interactions are also influenced by various factors in the external environment. Hence, when exploring biological evolution, we must also consider the interactions between the organism itself and its microbiome. This prompts us to follow the “extended phenotype” theoretical framework in evolution [76] to study their coevolution and the effects of natural selection driving forces.

To integrate the rapidly emerging, fragmented knowledge of microbiome involvement in immune system modulation, nutrient metabolism, neurological functions, and so forth, is a major challenge for reevaluating traditional biological paradigms that focus solely on the host organism. The integration of evolution, ecology, the microbiome, biology, and the One Health paradigm not only reshapes the first principles of biology but also propels us toward unlocking the Holy Grail of understanding life [77, 78, 79]. The traditional self‐based theory of biology has expanded to embrace a population‐ and ecology‐based framework [12, 80], which will guide future research endeavors to unravel the intricacies of living organisms and ecosystems in ways previously unimaginable [25, 81].

### Microbiome‒host intrinsic interactions for life

Microbiome research extends its influence beyond individual organisms to entire ecosystems and has revolutionized our understanding of genetics. In addition to the traditional focus on host genetics, the intricate interplay between host and microbial genomes reveals a more nuanced genetic landscape [82, 83]. The microbiome contributes to the genetic diversity of ecosystems, influencing traits and characteristics beyond what can be attributed solely to host genes, which is the key to unraveling mysteries related to adaptation, evolution, and the genetic basis of complex traits [20, 84, 85]. For example, the microbial‐host‐isozyme dipeptidyl peptidase produced by some specific *Bacteroides* spp. in the gut microbiota was found to reduce active glucagon‐like peptide‐1 [86], which plays a role in regulating glucose metabolism. A novel 3‐succinylated cholic acid produced from the metabolism of host bile acids by *Bacteroides uniformis* was recently identified and found to be associated with liver damage in patients with metabolic dysfunction‐associated fatty liver disease [87]. The discovery of associations between the gut microbiota, such as *Faecalibacterium prausnitzii* and *Collinsella aerofaciens*, and human *ABO* and *FUT2* genetic profiles through the N‐acetylgalactosamine metabolic pathway [88] highlights the importance of genetic connections spanning the human genome and the bacterial metagenome, offering a valuable understanding of the interplay between the host and its microbiome.

Microbiome research, along with One Health principles, will also help us develop personalized medicine and therapeutics and effective countermeasures for disease prevention and public health [25, 81]. Microbiome‐based diagnostics and interventions have the potential to revolutionize healthcare [89, 90], moving from a one‐size‐fits‐all approach to precision medicine that considers the unique microbial signatures of individuals. Understanding how the microbiome contributes to immune system function, pathogen resistance, and overall health empowers us to develop preventive strategies. Microbiome‐based interventions, such as probiotics and microbial therapies, hold promise for mitigating the risk of infectious diseases and chronic conditions [81, 91].

### Next step for integrating microbiome and one health

**Figure 5:**
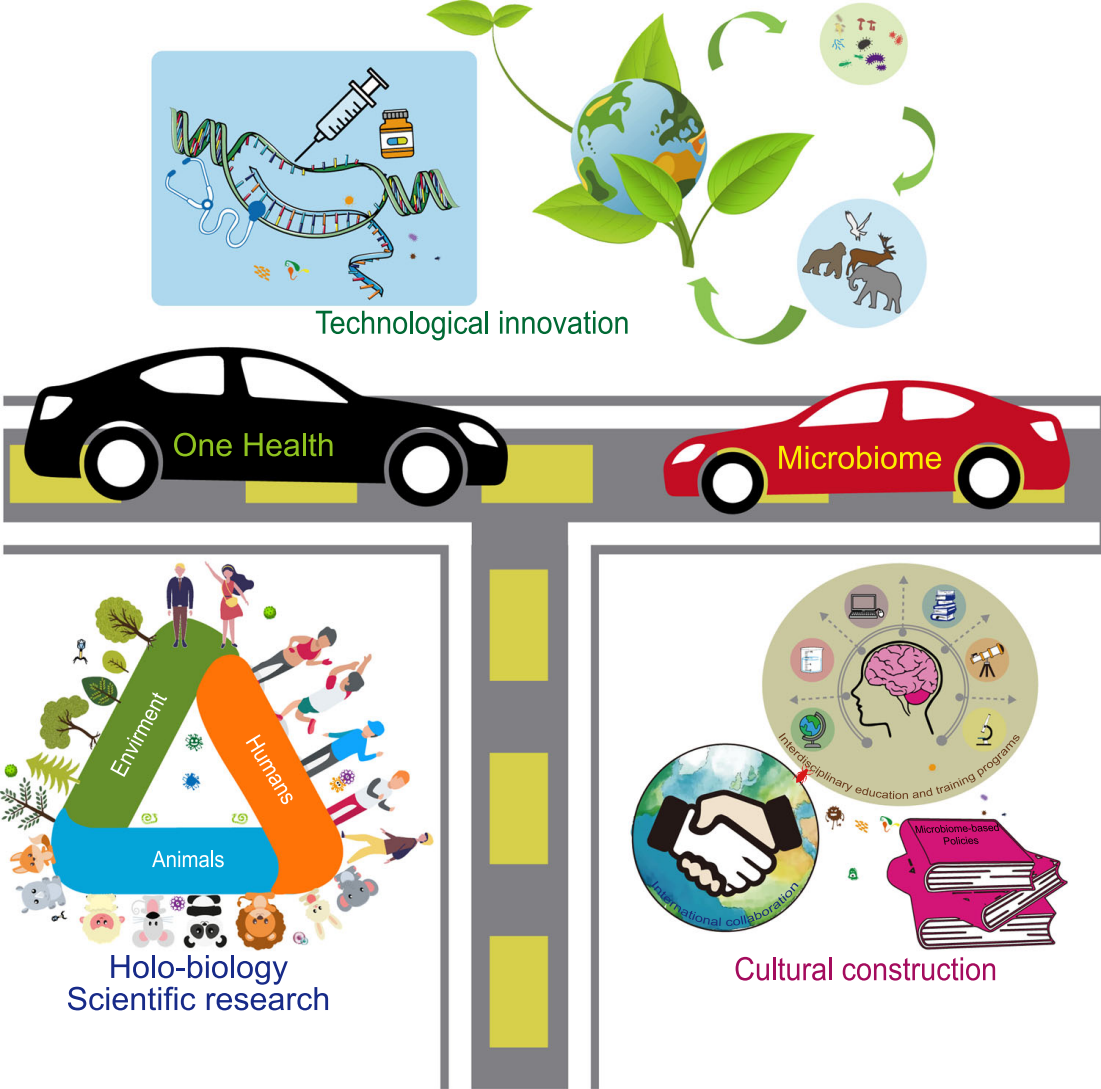
Next step for integrating the microbiome and One Health. The basic research of interdisciplinary studies, innovative technologies, and the cultural construction of the One Health concept, which is based on the microbiome, are three key aspects that promote the effective integration of the microbiome and One Health.

The integration of microbiome science with the One Health principle is not just a theoretical advancement but a practical imperative that promises to redefine the landscape of health for humans, animals, and the environment. As we stand at the crossroads of these disciplines, the path forward is illuminated by several key actions that shape the trajectory of this integration (Figure 5).

First, the basic research of interdisciplinary studies urgently needs to be strengthened to reveal how microecology mediates the balance of One Health and how the imbalance of the microbiome affects the health of humans, animals, and the environment. A holo‐biology based on our understanding of holobionts must be developed to reveal the science backing up microecology‐based One Health [92, 93, 94]. For the specific actions of interdisciplinary cooperation, the first step is that scientists from different disciplines, based on their mutual understanding of the scientific issues in their respective fields, have a common interest and enthusiasm in solving scientific problems that can be addressed only by interdisciplinary means. Thus, they must obtain project support to enable the project to be launched and implemented. During the implementation process, they have no selfish desire to obtain benefits from the project but focus only on solving scientific problems. For example, as mentioned earlier, scientists from different disciplines closely collaborated to reveal the possible reasons for the absence of natural plague foci in Europe both presently and historically [72].

Second, based on basic research, innovative technologies should be developed, such as technologies for assessing the balance and imbalance of the microbiome, technologies for restoring the balance of the microbiome, and technologies for maintaining balance. Among them, assessment and diagnostic technologies based on microecology should be prioritized for development. This enhancement will sharpen our early warning systems for disease detection and tailor treatment strategies to individual needs, a quantum leap from the generic to the bespoke. The crux of this advancement hinges on the adept application of big data analytics and artificial intelligence, which are essential for sifting through the genomic deluge to uncover the subtleties of microbial signatures. The realm of microbiome‐based therapeutics is set to expand the horizons of medical innovation [95]. From the rich tapestry of beneficial microbes and their metabolic byproducts, we can weave new treatments for a spectrum of diseases, offering hope from the common to the chronic. The validation of these therapeutic modalities through clinical trials will support their use in the medical arsenal against disease.

Third, it is necessary to strengthen the cultural construction of the One Health concept, which is based on the microbiome; to formulate interdisciplinary education and training programs, which are based on the balance of the microbiome; to carry out international cooperation for our common home on Earth; and to truly implement the concept and policies of One Health to maintain the common welfare of humans, animals, and the environment and to sustain the development of society [96]. The integration of microbiome science into public health policies is imperative. The cultivation of interdisciplinary education and training programs is vital to prepare a new cadre of professionals who can navigate the One Health paradigm. These educational initiatives should underscore the inseparable threads that connect human, animal, and environmental health, with the microbiome as the common weave. By forging alliances among researchers, healthcare systems, and governments, we can mount a collective defense against the rising tide of diseases, antimicrobial resistance, and environmental degradation. The exchange of knowledge and best practices will be the lifeblood of this global partnership. It is a call for policymakers to not only recognize but also legislate the importance of microbiomes in health and the environment. This includes establishing guidelines for probiotics, prebiotics, and fecal microbiota transplantation and formulating regulations to combat antimicrobial resistance.

In the recent past, several studies have underscored the importance of microbiome research and its integration with One Health principles. For instance, a study highlighted the host genetic regulation of human gut microbial structural variation, emphasizing the personalized nature of microbiome interactions [88]. In this report, the authors conducted a large‐scale meta‐analysis of genetic associations between human genotypes and microbial structural variations in the gut microbiome, encompassing 9015 individuals from four Dutch cohorts. Bioinformatics analysis and experimental validation revealed the causal genes involved in host‒microbiome interactions, enhancing our understanding of the regulatory role of genetic diversity in the gut microbiota in human genetics [88]. Another report indicated that the enrichment of beneficial bacteria, including *Nocardioides*, *Brevundimonas*, and *Sphingomonas*, in the rhizosphere microbiota helps pak choi plants resist pesticide stress, promoting plant growth while breaking down pesticide molecules [97]. Understanding the interactions among the root microbiota, pesticides, and plants will help us design novel strategies for pesticide application and chemical‐induced stress mitigation. Research into the extended microbiome and its metabolic implications has opened doors to understanding the profound impact of fermented foods on health [91]. These studies, along with others, form the foundation for future research directions and practical applications in the field.

As we venture further into the integration of microbiome science with the One Health principle, our focus on precision diagnostics, therapeutic innovation, policy advocacy, interdisciplinary learning, and international synergy will not only chart a course toward a healthier future but also ensure the sustainability of our collective ecosystem. This is the next step in an evolutionary journey that respects the dynamic interplay of life and the environment, promising a future where health and well‐being are in harmony with the natural world.

## 6 LIMITATIONS OF THEORY “BALANCE OF DYNAMIC FACTORS”

The concept of “balance of dynamic factors” within the framework of the microbiome and One Health indeed presents a holistic view of the interconnectedness of human, animal, plant, and environmental health, emphasizing the role of microbiomes as critical links for sustainable development. However, like any scientific theory, it faces challenges related to complexity, definitional clarity, temporal and spatial dynamics, anthropogenic influences, coevolutionary processes, technological limitations, practical application, and interdisciplinary collaboration. Addressing these limitations will be crucial for advancing our understanding and application of this theory in promoting health and sustainability.

The limitations of this theory include the following: (1) The complexity and heterogeneity of microbiomes make their combination with the One Health principle incredibly diverse and dynamic, varying across different hosts and environments. The “balance of dynamic factors” may not fully capture the complexity of these interactions because of the vast diversity of microbes and their functions. (2) Lack of a unified definition of “balance of dynamic factors” causes issues in establishing a universally accepted framework for the theory. (3) Human activities can significantly alter microbiomes, sometimes leading to imbalances. The theory needs to consider the impact of these changes on the balance it seeks to maintain, especially with the increasing recognition of the role of microbiomes in planetary health. (4) Microbes and their hosts are engaged in a continuous process of coevolution. The “balance of dynamic factors” might not fully encapsulate the evolutionary processes that drive these relationships and their impact on overall health and sustainability. (5) Advances in microbiome research are often driven by new technologies, which can introduce biases or require new definitions and standards. This can make it difficult to apply the theory consistently across different studies and environments. (6) While this theory provides a conceptual framework, its practical application in managing microbiomes for health and sustainability is still in its infancy. There is a need for more research on how to manipulate microbiomes effectively to achieve the desired balance. (7) The “balance of dynamic factors” cuts across various disciplines, requiring a high level of interdisciplinary collaboration. This can be challenging owing to differences in terminology, methodologies, and research objectives.

## 7 CONCLUSION

The intersection of microbiome science and One Health principles fosters balanced microbial ecosystems, resilience in health systems, and sustainable practices that benefit global health and social sustainability. The development of a universal balance theory “Balance of Dynamic Factors” for integrating these two fields has the potential to revolutionize global health and social well‐being for disease prevention, health equity, and sustainability. Embracing this integrated approach is essential for creating a healthier, more equitable, and sustainable future for generations.

## AUTHOR CONTRIBUTIONS

Ruifu Yang and Jing Yuan conceived this review and wrote the draft. Yazhou Zhou, Ziying Xu, Huan Zhang, Teng Liu, Jin Zhou, Yujing Bi, Yanping Han, and Yafang Tan were involved in the writing of this review. All the figures were prepared by Ziying Xu and revised by Ruifu Yang and Jing Yuan. All the authors have read the final manuscript and approved it for publication.

## CONFLICT OF INTEREST STATEMENT

The authors have declared no competing interests.

## ETHICS STATEMENT

No animals or humans were involved in this study.

## Data Availability

No new data and scripts were generated in this review. Supplementary information (graphical abstract, slides, videos, Chinese translated version, and updated materials) may be found in the online DOI or iMeta Science http://www.imeta.science/imetaomics/.
